# Simplifying models and estimating grasp performance for comparing dynamic hand orthosis concepts

**DOI:** 10.1371/journal.pone.0220147

**Published:** 2019-07-25

**Authors:** Ronald A. Bos, Dick H. Plettenburg, Just L. Herder

**Affiliations:** 1 Department of Biomechanical Engineering, Delft University of Technology, Delft, The Netherlands; 2 Department of Precision and Microsystems Engineering, Delft University of Technology, Delft, The Netherlands; Worcester Polytechnic Institute, UNITED STATES

## Abstract

While designing a dynamic hand orthosis to assist during activities of daily living, the designer has to know whether a concept will have sufficient grasp performance to support these activities. This is often estimated by measuring the interaction force at the contact interface. However, this requires a prototyping step and limits the practicality of comparing several concepts in an early design stage. Alternatively, this study presents and compares basic static and dynamic models to numerically estimate grasp performance. This was applied on an exemplary concept for a hydraulically operated hand orthosis grasping a circular object. The models were validated with an experimental set-up that does not require sensors at the contact interface. Static and dynamic model results were almost identical, where the static model could be around 10 times faster and is generally more robust to a high contact stiffness. Both models were unable to make accurate quantitative predictions, which is believed to be due to differences in used contact stiffness. However, the models were able to make correct qualitative comparisons, making it a valid method to compare and choose concepts in an early design stage.

## Introduction

Sometimes, an impairment can affect the neuromuscular function of our hands. For example, the available strength in the hand muscles can be limited by the effects of aging or a muscular disorder. When an individual loses the ability to grasp objects, it becomes more difficult to perform daily activities, maintain independence and participate in social activities. In order to recover the ability to grasp, additional strength can be added to the hand by using a dynamic hand orthosis that provides assistance during activities of daily living.

There are numerous dynamic hand orthoses being developed, with over 160+ examples over the past 50 years [1]. A certain diversity between these devices can be found in applications, system designs and testing procedures. Partly due to this diversity, there is also a wide range of methods to characterize the mechanical functioning of this type of device. For a daily assistive device, it is necessary to get an idea of the grasping performance. For example, a custom actuator can be evaluated by measuring actuator strength [2], a particular mechanism can be evaluated by measuring tip force [3], or the full orthosis design can be evaluated by having an individual wear the orthosis and measure grasping force with a sensorized object [4]. The advantage of these methods is that they provide a measure of actual performance and can be used to verify predictions. The disadvantage, however, is that they cannot be performed in an early design stage. Being able to estimate grasping performance during this stage can help with optimizing mechanism topologies and shapes. Because the early design stage is characterized by creating and weighing concepts, any step that requires manufacturing impedes the design process. To the author’s knowledge, there is currently no generalized method presented that is able to estimate grasping performance of a dynamic hand orthosis in this conceptual design stage.

The purpose of this study is to present a method that is able to estimate the grasping force using a model that does not require much a priori knowledge, which can be used as a tool to aid in choosing between conceptual designs. The intention behind this method is not to be anatomically correct, but to minimize the amount of details while preserving the ability to make appropriate design choices. This way, one can get an idea of the grasping performance in an early design stage without fabricating and testing a prototype, or requiring accurate (subject-specific) anatomical properties. Moreover, beyond this study, the use of modeling techniques allows us to extract even more information like possible range of motion of the anatomical joints or interaction forces between the finger and orthosis, which can be used to maximize freedom of movement or to minimize shear forces.

In order to estimate the grasping force, one needs to find a stable grasping configuration and investigate the contact forces on the object. There are multiple ways to determine this configuration and can be narrowed down to finding a position where the system is in equilibrium. In this study, a dynamic model and a static model are compared and their applicability is discussed. Both models require several iterations to find the equilibrium position. A dynamic model performs an integration over time and a static model uses a minimization procedure for each individual position. To validate both models and assess the effect of the additional assumptions that come with a static model, a prototype was made and tested. All methods were applied on an exemplary design of a hydraulically operated hand orthosis.

## System description

**Fig 1:**
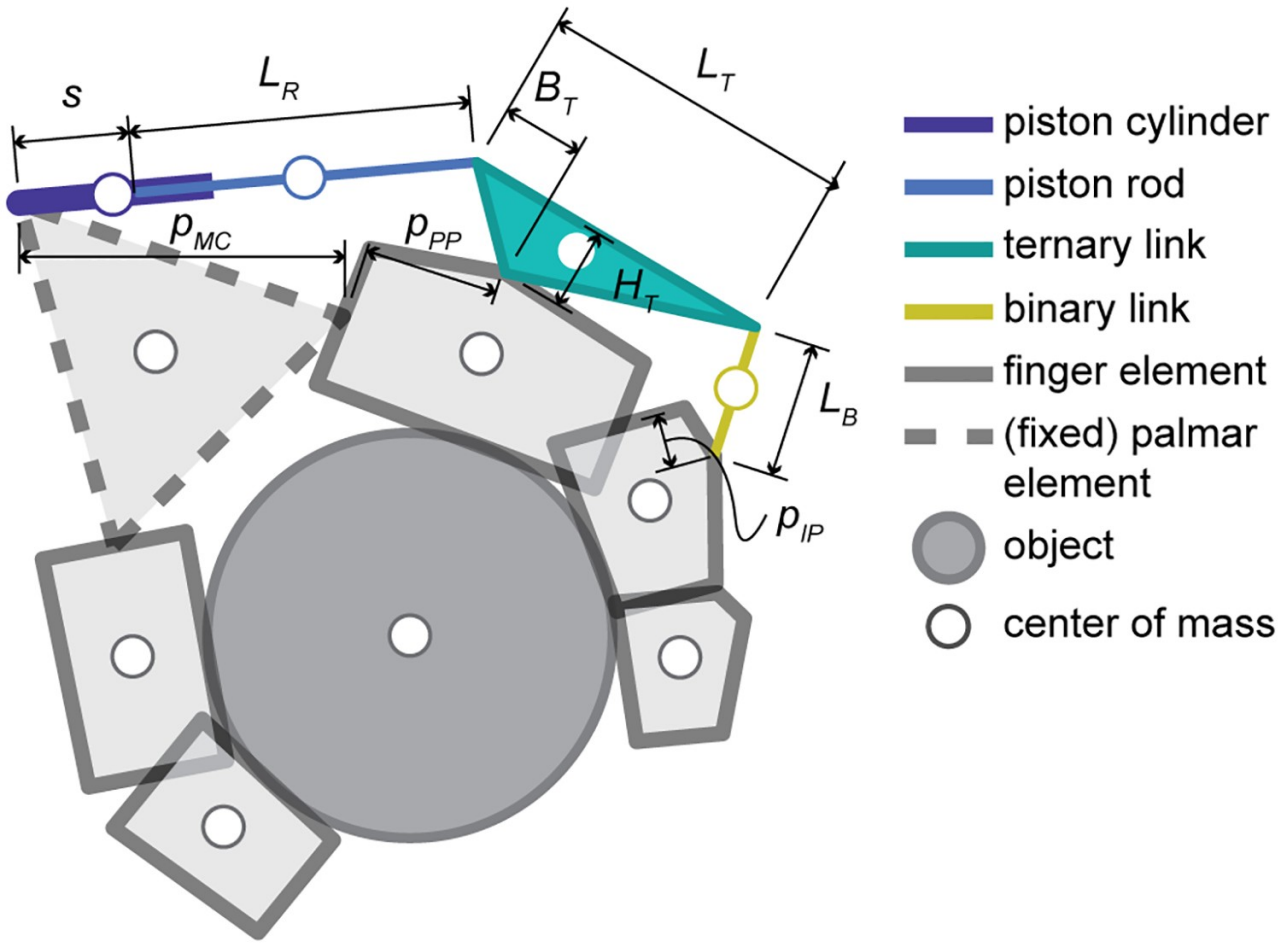
Basic illustration of the system as it appears in the model. Mechanism elements are indicated along with their lengths (*s*, *L*_*R*_, *L*_*T*_, *B*_*T*_, *H*_*T*_, *L*_*B*_) and locations of the interfaces at the phalanges (*p*_*MC*_, *p*_*PP*_, *p*_*IP*_). In this model, the length of the piston cylinder relates to the maximum stroke (*s*_*max*_) and the length of the piston rod (*L*_*R*_) equals the minimum distance between the hinge joints of the cylinder.

For the purpose of this study, a hydraulically operated hand orthosis mechanism was used. An illustration of the system is shown in Fig 1. A single hydraulic cylinder pushes a linkage mechanism with two degrees of freedom (DOFs), which then applies force to the proximal and intermediate phalange of the finger. Because the number of actuators is less than the mechanical DOFs, this is an underactuated mechanism and allows it to passively adapt to an object’s shape. The distal phalange is left free and does not actively contribute to grasping force during cylindrical grasps. Similar to the Delft Cylinder Hand [5], multiple finger mechanisms can be connected by a single master cylinder. This study, however, is limited to a two-dimensional scenario supporting only a single finger.

The shape of the linkage mechanism is based on the first isomer of a seven-link mechanism with two DOFs from Suarez-Escobar et al. [6]. A mechanism with only two DOFs was chosen, because a two-phalange system is still able to perform stable underactuated grasping [7] and avoids any physical connection between the mechanism and the distal phalange, which minimizes the risk of attenuating tactile feedback at the fingertip. This particular isomer was chosen based on the ability to easily replace a revolute joint by a prismatic joint (i.e., piston cylinder), while maintaining the capability to create a low-profile mechanism.

## Methods

### Ability to hold an object

As described in [8], a grasper can be assessed by calculating its ability to grasp and hold objects. In this context, the ability to grasp is defined by the geometry of the grasper, i.e., the number of phalanges and their lengths. A human hand is considered without limb-deficiencies in this study and so the ability to grasp is fixed. The ability to hold objects, on the other hand, remains variable. It is defined by the grasping force that the grasper is able to exert on an object. In [8], this was tested by determining the minimum force required to pull an object out of the grasper, in a direction that is perpendicular to the grasper’s axes of rotation. Although a very effective method for robotic graspers, the joint stiffness of a human hand can greatly affect this metric. This is because the hand is opened while the object is being pulled out of the grasp, introducing the risk to measure joint stiffness rather than grasping force. Hence, in this study, the object was pulled in a direction that was parallel to the joint axes. The ability to hold an object can then be defined by the sum of normal forces of the finger’s phalanges on an object at the instant when the grasp is stable. This way, the ability to hold an object is more dependent on the mechanism’s ability to transfer and distribute forces, and less dependent on the grasper’s inherent stiffness characteristics.

In order to assess the system’s ability to hold an object, the interaction with a graspable object needs to be modeled. This is preferably done without requiring a priori knowledge on the contact configuration. For example, when modeling one finger with three phalanges and one opposing thumb with two phalanges, this gives a total of six possible contact points (i.e., five phalanges and one palm element). If all possible configurations with at least two contact points need to be investigated, this results in ($\sum_{k=2}^{6}\frac{6!}{k!(6-k)!}=$) 57 different scenarios. Even though some of these configurations could be excluded, it would be much more efficient to let the model determine the stable grasp configuration.

In this study, the ability to hold an object is analyzed for an open fist cylindrical grasp [9]. This particular grasp was chosen to be representable for picking up objects with a circular cross-section (e.g., bottles, cans). Because this grasp holds the objects by form-closure, friction will not have an effect on the grasping shape. Neglecting static friction will then result in a worst-case scenario, where the actual grasping force can only be higher. Additionally, the thumb was simplified with only two phalanges and acting in the same plane as the finger. Although this does not reflect the anatomy of the human hand, this was not deemed necessary because forces acting on or inside the thumb were not of interest in this exemplary study. The simplified thumb element fulfilled the purpose of providing an opposing force and allowing analysis of the finger elements that interface with the orthosis mechanism.

### Contact model

In order to determine whether a contact point is active, the model needs to detect the distance between the potential contact points. Using this distance, the appropriate contact force can be applied using a contact force model.

#### Contact detection

The moment when the distance between two bodies becomes zero, they are in contact and the appropriate contact forces should be applied. Hence, contact is detected by monitoring the shortest distance between two potential contacting bodies. For the purpose of this study and similar to [8], only circular objects were considered and the contacting elements of the hand were represented by straight lines. This way, the shortest distance between the object and a hand element could be narrowed down to the shortest distance between a point and a line segment.

**Fig 2:**
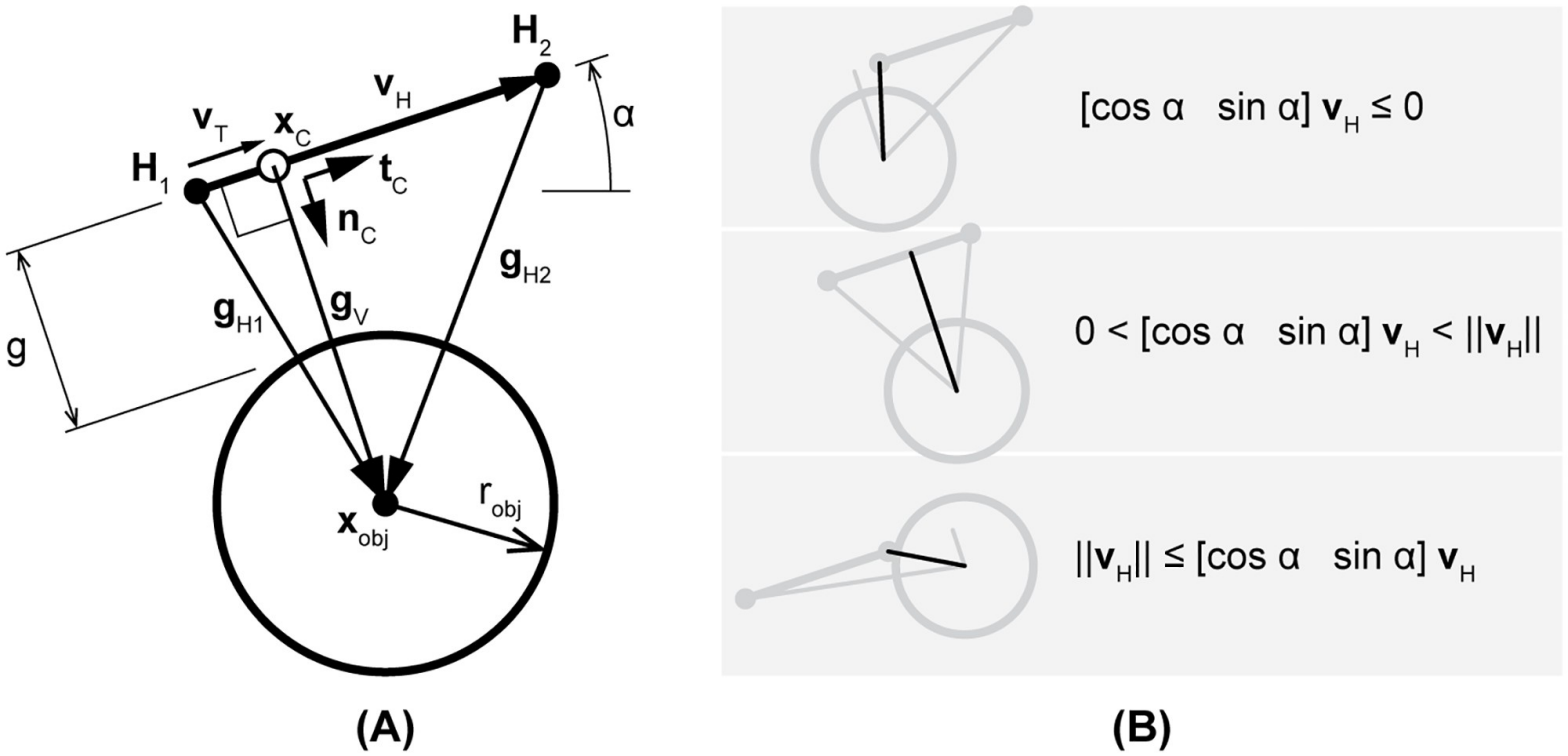
Determination of the shortest distance vector. (A) Potential distance vectors (**g**_*H*1_, **g**_*V*_, **g**_*H*2_) between a circular object (position **x**_*obj*_ and radius *r*_*obj*_) and line segment (defined by vector **v**_*H*_ with endpoints **H**_1_ and **H**_2_). In the shown configuration, vector **g**_*V*_ is shortest and creates a contact point (**x**_*C*_) at the line segment, along with local tangential and normal unit vectors (**t**_*C*_, **n**_*C*_). (B) Three different configurations that illustrate when which distance vector should be used to determine the shortest distance.

Fig 2A illustrates an arbitrary situation between two potential contacting bodies. It shows that, depending on the position of the circular object, three potential distance vectors can be defined. Using the symbols from Fig 2A, the distances between the object and the endpoints can be easily determined:

$$\begin{array}{c} \left|\right|g_{H1}\left|\right|=\left|\right|H_{1}-x_{obj}\left|\right| \end{array}$$ (1)

$$\begin{array}{c} \left|\right|g_{H2}\left|\right|=\left|\right|H_{2}-x_{obj}\left|\right| \end{array}$$ (2)

The distance vector **g**_*V*_, on the other hand, can be determined by using the projection of **g**_*H*1_ on **v**_*H*_, according to:

$$\begin{array}{c} \left|\right|g_{V}\left|\right|=\left|\right|g_{H1}-v_{T}\left|\right| \end{array}$$ (3)

where

$$\begin{array}{c} v_{T}=(g_{H1}•{\hat{v}}_{H}){\hat{v}}_{H} \end{array}$$ (4)

$$\begin{array}{c} {\hat{v}}_{H}=\frac{v_{H}}{\left|\right|v_{H}\left|\right|} \end{array}$$ (5)

To determine which distance vector should be used for the shortest distance at a particular instant, the location of the potential contact point (**x**_*C*_) needs to be determined which is constrained to be on the line segment defined by **v**_*H*_. If **x**_*C*_ appears to lie on or beyond the boundaries defined by endpoints **H**_1_ and **H**_2_, it means that **x**_*C*_ needs a correction towards the nearest endpoint. This can all be done by using the projection vector **v**_*T*_ and determining its length and orientation with respect to **v**_*H*_ (see Fig 2B), resulting in the following conditional equations for the distance between the object and line segment:

$$\begin{array}{c} g=\{\begin{array}{cc} \left|\right|g_{H1}\left|\right|-r_{obj} & \text{if}\;[\begin{array}{cc} \cos\alpha & \sin\alpha \end{array}]v_{T}\leq 0\\ \left|\right|g_{V}\left|\right|-r_{obj} & \text{if}\;0<[\begin{array}{cc} \cos\alpha & \sin\alpha \end{array}]v_{T}<||v_{H}\left|\right|\\ \left|\right|g_{H2}\left|\right|-r_{obj} & \text{if}\;||v_{H}\left|\right|\leq [\begin{array}{cc} \cos\alpha & \sin\alpha \end{array}]v_{T} \end{array} \end{array}$$ (6)

Additionally, by using Eqs 3–5, the coordinates of the contact point can be determined and a set of local unit vectors can be defined:

$$\begin{array}{c} x_{C}=H_{1}+v_{T} \end{array}$$ (7)

$$\begin{array}{c} t_{C}=\frac{v_{T}}{\left|\right|v_{T}\left|\right|} \end{array}$$ (8)

$$\begin{array}{c} n_{C}=\frac{g_{V}}{\left|\right|g_{V}\left|\right|} \end{array}$$ (9)

These expressions are particularly useful to apply the appropriate contact forces in the correct directions.

#### Contact force

In both the dynamic and static model, contact with an object was added by using a continuous (or compliant) model. This approach was chosen to be favorable over using a discrete model, which limits itself to rigid contacts. Especially in a dynamic model, this causes instantaneous changes in the system and becomes inconvenient in numerical integration with multiple possible contact points [10]. The continuous model, on the other hand, simulates a (visco-)elastic interface between the contacting bodies and allows to model flexible objects. Similar to Hertz’s contact theory, the indentation between two objects can be related to a contact force. In Machado et. al [11], several extensions to this model are discussed, including this dissipative contact force model:

$$\begin{array}{c} F_{N}=K_{c}\delta^{n}(1+\frac{3\left(1-c_{r}^{2}\right)e^{2(1-c_{r})}}{4}\frac{\dot{\delta }}{{\dot{\delta }}^{\left(-\right)}}) \end{array}$$ (10)

Here, *F*_*N*_ is the contact force perpendicular to the surface, *δ* the indentation, $\dot{\delta }$ the indentation velocity, ${\dot{\delta }}^{\left(-\right)}$ the initial velocity during contact, *K*_*c*_ the contact stiffness factor and *c*_*r*_ the coefficient of restitution. Assuming a parabolic pressure distribution, the power exponent (*n*) was taken as equal to $\frac{2}{3}$ [11].

This contact force model is particularly suitable for situations with a low coefficient of restitution [11], which is assumed to be the case in the presented situation of a hand grasping an object. The model was coupled with Eqs 1–6, by defining the indentation as the overlap between the contacting bodies:

$$\begin{array}{c} \delta =\min(g,0) \end{array}$$ (11)

It was assumed that the magnitude of indentation would not exceed the radius of the object. Because if *δ* > *r*_*obj*_, the direction of **n**_*C*_ would change and *F*_*N*_ will act in opposite direction. Even though this is unlikely to happen, it can produce invalid results when very small objects are chosen with a low contact stiffness.

### Dynamic model

When using a dynamic model, effects such as friction, viscosity and inertial forces can be implemented and the motion of the system can be investigated. The equilibrium position can be found by simulating a system over time until the accelerations approach zero. The basis of the dynamic model revolves around the equations of motion of a constrained system of rigid bodies. This combines Newton’s second law of motion with constraint equations by using the technique of Lagrange multipliers:

$$\begin{array}{c} \left[\begin{array}{cc} M & D_{q}^{T}\left(q\right)\\ D_{q}\left(q\right) & 0 \end{array}][\begin{array}{c} \ddot{q}\\ \lambda \end{array}]=[\begin{array}{c} f(\dot{q},q,t)\\ -g(\dot{q},q,t) \end{array}\right] \end{array}$$ (12)

The bodies’ states, velocities and accelerations are denoted by **q**, $\dot{q}$ and $\ddot{q}$, respectively. Values for mass and inertia are stored in the mass matrix, **M**. Contact forces, actuator forces and other viscoelastic elements were all applied as external forces that are stored in the force vector, **f**. Constraint equations were implemented using their jacobian, **D**_**q**_, and the convective terms that follow from the second order derivatives with respect to time, **g**. Lastly, the Lagrange multipliers are denoted by **λ** and can be interpreted as the magnitude of constraint forces. These equations can be used to evaluate the system over time using numerical integration, where the values of the Lagrange multipliers can be used to inspect the internal forces of the system.

#### Friction force

Friction is an extremely complex phenomenon and is strongly dependent on the types of materials that are interacting. Considering the high variability of graspable objects and dependency of the friction coefficient on numerous factors [12, 13], the friction forces from this model were not considered to be representative for a real-life situation. Nonetheless, friction was implemented to provide for additional numerical stability. More specifically, it was added to avoid undamped sliding of the object along the finger elements.

In the classic Coulomb model, the friction force is equal to a friction coefficient times the normal force. This force is then applied in opposite direction of the relative movement between the contacting bodies. This results in a sudden change in forces on the system, which can severely slow down the numerical integration process around these events. Instead, the friction force was defined with a smooth transition around close-to-zero velocities:

$$\begin{array}{c} F_{T}=\text{sgn}\left({\dot{t}}_{C}\right)\mu F_{N}\;\min(1,\frac{{\dot{t}}_{C}}{v_{T}}) \end{array}$$ (13)

Here, *F*_*T*_ represents the friction force which scales with the normal force according to the frictional coefficient, *μ*. Direction of the friction force is determined by the relative tangential velocity at the contact point between the two bodies, ${\dot{t}}_{C}$. For relative velocities below a characteristic velocity, *v*_*T*_, the friction force is scaled as a ramp function. The advantage of this method is that it stabilizes the numerical integration algorithm at low velocities compared to the classic Coulomb model [14]. However, it only models dynamic friction and neglects stick-slip effects caused by a higher static friction coefficient.

#### Numerical integration

The contact and friction force models conveniently provide a smooth transition from zero, thus giving a method suitable for numerical integration. Nonetheless, the sudden application of these forces still introduces an abrupt change in the system. For this reason, an event-detection scheme was used to detect the moment when a new contact force should be added (or an old one removed). This stops the integration process, adds the contact forces to the model, and continues the integration where it left off. This allows for larger time steps and overall faster computation. A standard solver (ode15s from Matlab) was used that is able to deal with stiff equations. To further speed up the numerical integration process, the functions that generate the matrices for Eq 12 were converted to faster MEX-files.

### Static model

As opposed to the dynamic model, a static model neglects inertial effects and makes accelerations of bodies obsolete. Instead of simulating the system over time, it already assumes that accelerations are zero and finds the appropriate system configuration. This configuration can be found by performing a constrained minimization procedure. In particular, it minimizes the total potential energy of the system according to:

$$\begin{array}{c} \begin{array}{cc} \min_{x}f\left(x\right)= & \sum_{e=1}^{n_{e}}\int F_{e}\left(x\right)ds_{e}\left(x\right)-\sum_{a=1}^{n_{a}}\int F_{a}\left(x\right)ds_{a}\left(x\right)\\ \text{s}.\text{t}.:\; & D_{eq}\left(x\right)=0\\ & D_{ineq}\left(x\right)\leq 0 \end{array} \end{array}$$ (14)

In this expression, the elastic energy stored in all *n*_*e*_ elastic elements increase the potential energy, which also include the Hertzian contacts with the object. The work done by all *n*_*a*_ actuating elements decrease the total potential energy. The shape of the mechanism was represented by constraint equations. These can be equality constraints, *D*_*eq*_, similar to what was used in the dynamic model, as well as inequality constraints, *D*_*ineq*_. Lastly, the **x**-vector contains the leftover degrees of freedom that are not connected by the constraint equations. In this study, these are the actuator strokes, finger joint angles and object position. By varying these variables and calculating their corresponding potential energies, a solution can be found within the constraints where the potential energy is minimal.

In contrary to the dynamic model, the minimization procedure allows for an easier implementation of inequality constraints. This provides for a convenient way to, for example, limit an actuator’s stroke or prevent links from the mechanism to penetrate finger elements. In a dynamic model, these need to be implied using contact models or non-smooth unilateral constraints.

Similar to the dynamic model, Lagrange multipliers can be used for all equality constraints to inspect the internal forces of the system. If the same constraint equations are used from the dynamic model, Eq 12 can be used with $\ddot{q}=0$:

$$\begin{array}{c} D_{q}^{T}\lambda =f(\dot{q},q,t) \end{array}$$ (15)

$$\begin{array}{c} D_{q}D_{q}^{T}\lambda =D_{q}f\left(\dot{q},q,t\right) \end{array}$$ (16)

$$\begin{array}{c} \lambda ={\left(D_{q}D_{q}^{T}\right)}^{-1}D_{q}f\left(\dot{q},q,t\right) \end{array}$$ (17)

#### Friction force

Because friction is not a conservative force, it does not add to the total potential energy of the system and was therefore not included.

#### Minimization procedure

Because the minimization function and the constraint equations are highly non-linear, a robust minimization algorithm is needed. For this reason, the SQP-algorithm stored in Matlab’s fmincon function was chosen. All constraint equations where added as non-linear equality and inequality constraint functions. To speed up the algorithm, variables and results from the objective function (*f*(**x**)) were shared with the constraint function expressions.

For different initial conditions, the SQP-algorithm can find different minima. Especially the initial guess for object position had a large influence on which local minimum was found by the algorithm, which could also be outside the grasp. To ensure that the algorithm finds the minimum that corresponds to a stable grasp, a range of initial object positions was used. This range was defined by a 15 mm square grid that fell within the possible range of motion of the hand. The solution with the lowest gradient around the found minimum was then selected, as this would represent the most stable solution.

### Experimental set-up

**Fig 3:**
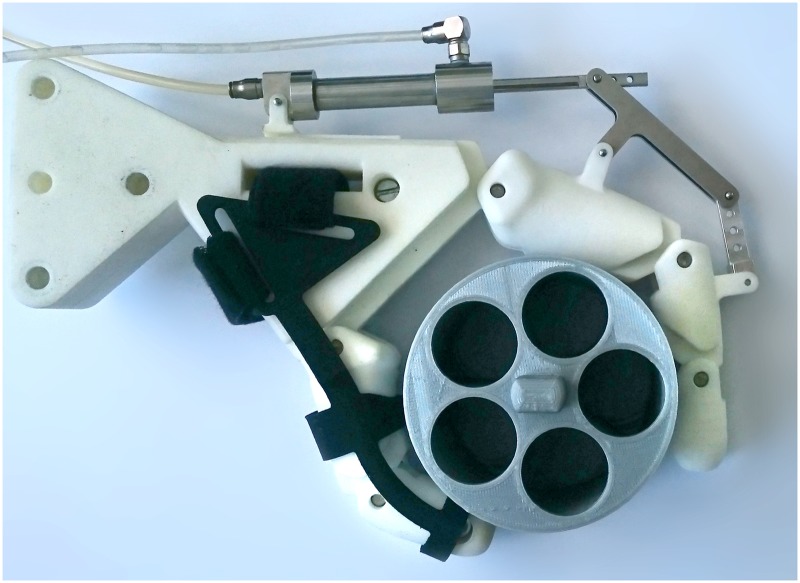
Picture of the manufactured prototype fixed on a mock-up hand, holding a circular object. The object was pulled out of the grasp with a cable connected to a force sensor. The hydraulic cylinder was connected to a master cylinder, onto which a weight was suspended for a constant system pressure.

In order to test the model outputs to a real-world situation, the ability to hold a circular object was measured using a prototype version of the hand orthosis system (see Fig 3). The prototype was fitted onto an anthropomorphically shaped mock-up hand, consisting of one finger and one opposing thumb. In this mock-up hand, the joints were fitted with torsional springs with a low stiffness (0.07 Nm/rad) in order to simulate a slightly flexed resting position of the phalanges. The thumb was fixed in opposition by strapping a 3D-printed splint onto the mock-up hand. The dimensions and material of this splint added a bending stiffness of approximately 8 Nm/rad parallel to each thumb joint. Fixation points were attached to the mock-up hand and facilitated a connection with the orthosis mechanism. These fixation points provided for a rigid connection between the mechanism and the finger, which is similar to the model representation.

In order to test the sensitivity of the mechanism to changes, the link length of the ternary link (*L*_*T*_) and object diameter (*D*_*obj*_) was varied. This simulated a crude sensitivity analysis and allowed to analyze how the outcome measure was affected by changing these parameters. The mechanism’s shape was therefore varied with only one dimension, to prevent having to perform countless experiments and to maintain the ability to easily visualize the results. Naturally, a more realistic shape optimization scheme would include all key mechanism dimensions along with a sensitivity analysis around the optimum.

Changing *L*_*T*_ was expected to alter the range of motion and moment arm around the finger’s proximal interphalangeal joint, affecting the attainable grasping force. Changing *D*_*obj*_ would test the mechanism’s robustness to grasping different sizes of objects. The variations that were applied, along with the model and prototype dimensions, are shown in Table 1. In this table, hand interface locations describe the positions where the orthosis mechanism physically connects with a finger metacarpal or phalanx.

**Table 1:** Dimensions used for modeling and construction of the mechanism.

Variables		
Dimension	Symbol	Variations [mm]
Ternary link base length	*L*_*T*_	40, 50, 60
Object diameter	*D*_*obj*_	60, 67.5, 75
Parameters		
Dimension	Symbol	Value [mm]
Mechanism dimensions		
Cylinder maximum stroke	*s*_*max*_	36
Cylinder bore diameter	*D*_*c*_	8
Piston rod length	*L*_*R*_	63
Ternary link base position	*B*_*T*_	15
Ternary link height	*H*_*T*_	15
Binary link length	*L*_*B*_	25
Hand dimensions		
Proximal phalange length	*L*_*PP*_	55
Intermediate phalange length	*L*_*IP*_	33
Distal phalange length	*L*_*DP*_	26
Palm element length	*L*_*P*_	60
Thumb proximal phalange length	*L*_*tPP*_	43
Thumb distal phalange length	*L*_*tDP*_	33
Proximal phalange thickness	*t*_*PP*_	25
Intermediate phalange thickness	*t*_*IP*_	19
Distal phalange thickness	*t*_*DP*_	16
Thumb proximal phalange thickness	*t*_*tPP*_	26
Thumb distal phalange thickness	*t*_*tDP*_	22
Hand interface locations		
on metacarpals	*p*_*MC*_	60
on proximal phalange	*p*_*PP*_	25
on intermediate phalange	*p*_*IP*_	10

The objects were hollow cylinders and constructed using 3D-printed PLA. A diameter of 67.5 mm coincides with the average diameter of over 70 different 16 oz./500 mL bottles of popular brands [15] and can be associated with an object mass of approximately 500 g. To accommodate this size and a range of 300-700 mL bottles as well, object diameters were varied between 60, 67.5 and 75 mm. All cylindrical objects had a height of 55 mm. Because the largest object had a mass of 80 g, gravitational effects were neglected.

The contact interaction between the 3D-printed mock-up hand and objects was considered as a stiff contact with a low coefficient of restitution (*c*_*r*_ = 0.1). The contact stiffness in the models was chosen as high as possible while maintaining a stable algorithm, which was mostly limited in the dynamic model. As a result, the dynamic model used a stiffness factor of $K_{c}=1\cdot 10^{5}\;{\text{Nm}}^{\frac{2}{3}}$ (see Eq 10), whereas the static model was able to use a value of $K_{c}=1\cdot 10^{9}\;{\text{Nm}}^{\frac{2}{3}}$. The fact that the models use different values for contact stiffness to simulate a rigid contact, is considered to be part of the models’ traits and therefore part of the comparison between the modeling approaches.

Pressure on the cap-side of the hydraulic cylinder was applied by loading a master cylinder with a mass, until a pressure of 1.25 MPa was measured with a pressure sensor (SPAW-P50R-G12M-2PV-M12, Festo). A test bench was used to pull on the object in a direction that was horizontal and parallel to the joint axes, where force (*F*_*pull*_) was measured using a load cell (B3G-C3-50kg-6B, Zemic Inc). The object was pulled over a distance of at least 40 mm without losing contact with the object. In order to obtain the ability to hold the object (*F*_*hold*_), this pulling force was divided by the friction coefficient between the object and mock-up hand. Vice versa, the inverse operation could be applied to estimate *F*_*pull*_ from the model outputs.

To avoid stick-slip effects that may influence the results, the object was pulled out at a constant velocity and the mean force was extracted. Furthermore, each configuration was measured five times. The dynamic friction coefficient was determined by measuring the average force necessary to move a 2, 4, 6 and 8 kg mass at the same velocity, resulting in a measured friction coefficient of 0.12.

In addition to the grasping force, the torque ratio between the proximal interphalangeal and metacarpophalangeal joint (*M*_*PIP*_/*M*_*MCP*_) was calculated from the contact forces to accompany the model results. This provided additional information on how the orthosis mechanism distributed force over these two phalanges. All contact forces should be similar for an equal force distribution [16] and so the ideal torque ratios can be scaled according to phalange lengths [8], which is approximately 0.52 for this situation.

## Results

**Fig 4:**
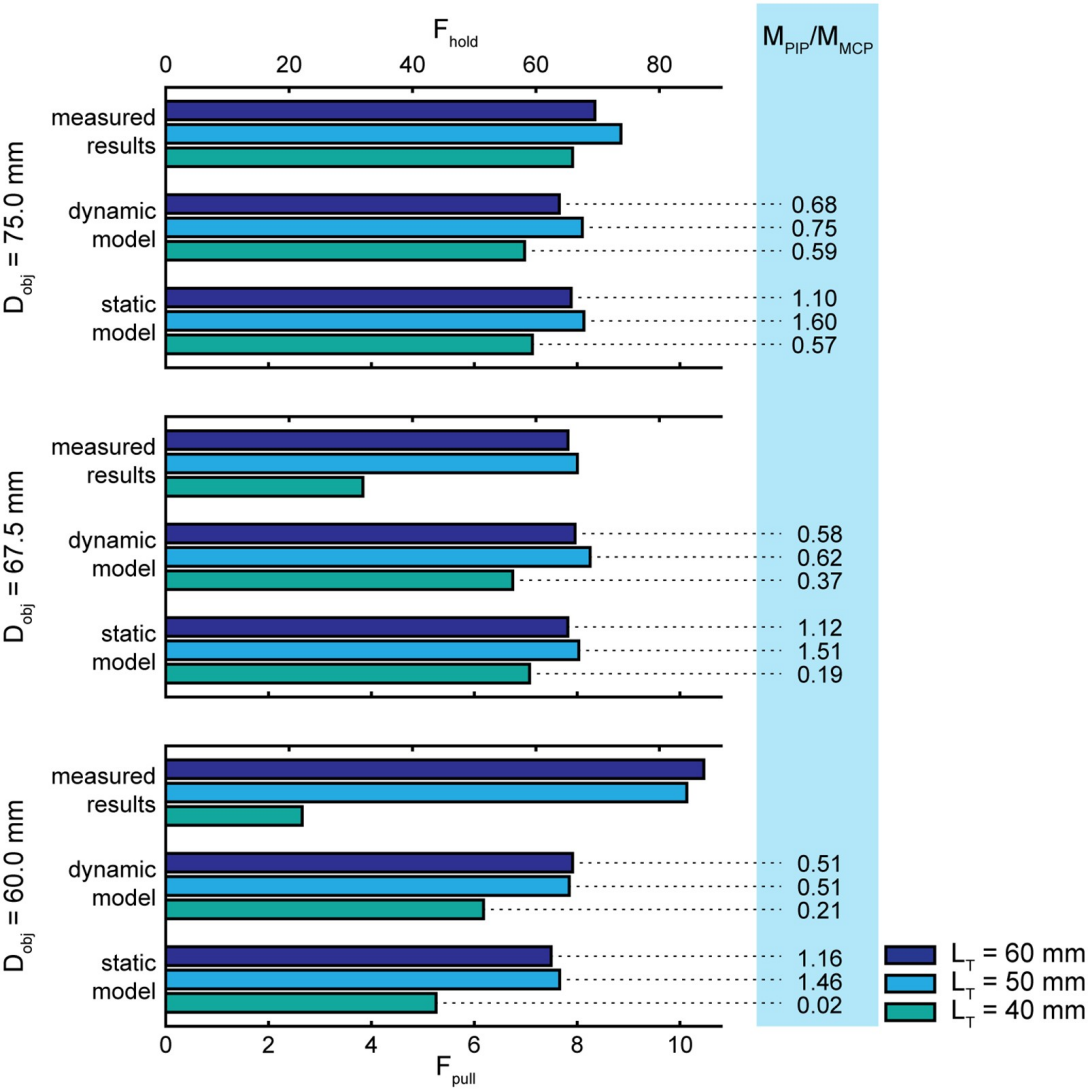
Measured and modeled results for forces related to the ability to hold objects. The sum all of normal forces (*F*_*hold*_) is shown on the top horizontal axis. The force necessary to pull an object out of the grap (*F*_*pull*_) is shown on the bottom axis. Additionally, the torque ratios (*M*_*PIP*_/*M*_*MCP*_) that accompany model outputs are displayed on the right.

The results for the force related to the ability hold objects are shown in Fig 4. Calculation times for the static model varied between 39–95 s, for the dynamic model this varied strongly between 118–1769 s. The results indicate, assuming a friction coefficient of 0.12, that *F*_*hold*_ for this mechanism ranged between 22–87 N according to the measured results, between 51–69 N according to the dynamic model predictions and between 44–68 N according to the static model predictions. The quantitative differences imply an agreement between the dynamic and static model, but a disagreement between these modeled outputs and the measured results.

Concerning relative differences, the outputs from the different methods (i.e., the measurements and models) agree within each object diameter. For instance, assuming *D*_*obj*_ = 67.5 mm, an optimization procedure would conclude with every method that *L*_*T*_ = 50 mm would give the highest possible grasping force. Compared to the measured results, however, the relative differences are very small between the model outputs.

In the static model, the torque ratios show more contrast than in the dynamic model. At *L*_*T*_ = 40 mm and *D*_*obj*_ = 60 mm, these torque ratios are (close to) zero for both models. This was also confirmed during the corresponding measurements, where it was observed that the intermediate phalange did not make contact with the object. Lastly, to illustrate the magnitude of the dynamic effects in the dynamic model, the individual masses of the finger elements were not larger than 6 grams and linear velocities of their centers of mass rarely exceeded 0.5 m/s.

## Discussion

### Dynamic versus static

In contrary to the dynamic model, the static model has no memory of previous states and can actually reach solutions that are not possible to reach or, in some cases, instable. A comparison of the outcomes of both models validates whether the static model is sufficiently constrained and able to reach the same conclusions as the dynamic model.

The results show that the grasping forces from the dynamic and static model are almost identical. This implies that the occurring velocities and inertial effects are indeed small enough to be neglected. The torque ratios, however, show more variations. More specifically, the qualitative differences between these ratios are similar, but the contrast is increased in the static model. Because the static model used a higher value for contact stiffness, we argue that a compliant contact interface is more likely to equally distribute forces than a rigid contact interface.

In general, the torque ratios slightly increase towards a larger object size. This means that the emphasis shifts from the proximal phalange to the intermediate/distal phalange as the object diameter increases, which is more similar to human grasping [17, 18]. However, comparison with existing literature is problematic because it usually describes grasping with a human hand [17] or a (symmetric) robotic grasper [7, 19]. This study, on the other hand, uses only the first two phalanges of a human finger and a passive distal phalange. Moreover, the range of ratios that provide stable grasping strongly depends on the geometry of the grasper and several different grasping types are possible in each range [7]. Therefore, in order to make more sense of these results, a grasping stability analysis is required for this particular situation.

### Model agreement

Despite the agreement between the dynamic and static model, the measured results show larger quantitative differences and especially towards smaller object sizes. We attribute these differences to small dissimilarities in dimensions and contact stiffness.

While configuring the models, it was found that both approaches were sensitive to small changes in the hand dimensions. The experimental situation differed slightly from the model representation and may therefore account for a portion of the disagreement. The model used conical cylinders whereas the mock-up hand used more curved shapes to approximate an anthropomorphic shape.

The contact stiffness with the object was not adjusted to the experimental situation, which required a very high stiffness value in order to approach the stiff contact. Especially the dynamic model was not able to handle high values for contact stiffness (*k* > 1 ⋅ 10^5^), because this quickly resulted in the numerical integration algorithm to become instable (e.g., due to close to singular matrices). Similar to a previous argument, we believe that a rigid contact amplifies the contrast in the outcome measures, because it becomes less likely that multiple phalanges can contribute to the grasping force. This can most easily be visualized by referring to the results where *D*_*obj*_ = 60 mm and *L*_*T*_ = 40 mm. The results with the most rigid contact (i.e., measured results) showed no contact at all with the intermediate phalange and a low grasping force, whereas the most compliant contact (i.e., dynamic model) showed a more distributed pattern and higher grasping force. Alternatively, at *D*_*obj*_ = 75 mm, the hand is better able to envelop the object and make contact with all phalanges, reducing the dissimilarities between the different methods.

### Orthosis mechanism evaluation

It was found that the orthosis mechanism was able to achieve a stable cylindrical grasp for all configurations. From the results, it can be concluded that the mechanism configuration with *L*_*T*_ = 50 mm provided the highest overall grasping forces at a pressure of 1.25 MPa. The estimated grasping forces for this mechanism configuration are high, ranging between 22–87 N. Considering that a force of 25 N between the thumb and fingertips is sufficient to lift a 2.25 kg object of similar size [20], the presented orthosis mechanism is able to exceed the requirement to facilitate grasping of objects in daily living.

### Generalization

Based on the presented system and experimental set-up, results cannot be generalized towards a situation that incorporates an anatomically correct human hand. However, conceptual designs can be compared in a qualitative way as they rely on the same simplifications. The connection with an anatomically correct hand is maintained by allowing the implementation of parametric stiffness characteristics in the anatomical joints and contact interfaces. Here, the static model allows for more freedom than the dynamic model, as it is not limited by stability issues that arise from the numerical integration process. By adding more details to the model, more similarities with the anatomical hand are introduced and quantitative outcomes are likely to further approach reality. For example, expanding to three-dimensional models can be helpful when other types of joints (e.g., spherical joints) are to be investigated, when a anatomically correct thumb is desired, or when multiple fingers are involved while grasping non-prismatic objects. Other pathological properties (e.g., stiff joints due to contractures) can be included in the model as well, for example by implementing joint stiffness profiles. However, further research is needed to investigate how such properties can affect the outcome of the model.

Beyond this particular example of a hydraulic hand orthosis with circular objects, the method presented in this study can be generalized towards a broader range of applications. The method is not limited by the use of hydraulic actuators, number of DOFs, object shape, two-dimensional scenarios or by hand orthoses. It allows any prescribed force, torque, translation or rotation to be used as an actuator, it allows any number of link elements to be connected by various joints and other convex-shaped objects can be used with the same contact detection scheme. When the mechanism becomes underactuated, however, it is important that enough stiffness elements (e.g., anatomical joint stiffness) are added to create a single valid equilibrium position.

The possibilities of the models can be further extended if the mechanism’s bodies, constraints and their connectivity are represented by parameters that are irrespective of the topology. Potential applications are topology optimization or even automated mechanism design [21]. Furthermore, flexible structures can be modeled using pseudo-rigid body modeling, but are more limited in accuracy. Addition of static friction can also include the analysis of grasps that are not bound by form closure.

The procedure to measure the grasping performance is simple and does not require an object to be equipped with pressure or force sensors. Therefore, any object can be pulled out of a hand that is fixed in position. This opens possibilities to validate the model predictions with human testing as well, which may also answer questions regarding compliancy of the contact interfaces.

## Conclusion

The results from this study show that a static model provides for a fast and adequate alternative to a dynamic approach to investigate the ability to hold circular objects. Less assumptions are required in order to estimate parameters related to dynamic effects (e.g., viscosity), rigid mechanical stops can be easily combined with compliant contact models and computation times can be around 10 times faster. However, there remain some discrepancies with experimental results that question the applicability of both models for real-world situations. These dissimilarities are mostly attributed to small differences in the used contact stiffness, where expansion to human testing can be the best method to determine model validity. Nonetheless, when relying on qualitative comparisons instead of quantitative predictions, the static model can be used to compare potential designs or even in optimization. Especially in an early design stage this is a useful tool for making well-supported design choices.

## Supporting information

S1 File Dataset. Matlab *.mat-file, which includes a structure that contains the simulation output data (e.g., grasping force, torque ratio, joint angles, simulation time) and measured values from the experimental set-up (average and minimum/maximum grasping force).
(MAT)

S2 File Figure generator. Matlab *.m-file, which uses the Dataset file and generates the results figure as displayed in this paper.
(M) Click here for additional data file.
